# Changes in C-Reactive Protein Forecast Infection Risk Following Treatment with Chimeric Antigen Receptor T Cells

**DOI:** 10.3390/cancers18182915

**Published:** 2026-09-09

**Authors:** Poonam Mathur, Hegang Chen, David J. Riedel, Elizabeth Holland, Danica Palacio, Jacqueline T. Bork, Katya Prakash, Nancy Hardy, Mehmet Kocoglu, Ashraf Badros, Ariel Fromowitz, Jean Yared, Aaron P. Rapoport, Djordje Atanackovic, John Baddley

**Affiliations:** 1 Institute of Human Virology, University of Maryland School of Medicine, Baltimore, MD 21201, USA; 2 Perelman School of Medicine, University of Pennsylvania, Philadelphia, PA 19104, USA; 3 Zucker School of Medicine, Northwell Health, Manhasset, NY 11030, USA; 4 University of Maryland Greenebaum Comprehensive Cancer Center, Baltimore, MD 21201, USA; 5 Department of Medicine, Johns Hopkins University School of Medicine, Baltimore, MD 21287, USA

**Keywords:** CAR-T, infection, CRP

## Abstract

Background. Immunosuppressive conditioning regimens for chimeric antigen receptor-modified T cell immunotherapy (CARTx) and subsequent T cell dysfunction increase risk for infection. However, it is difficult to discern infection from CRS in the early (up to Day 30) and late (Days 31–180) periods after CARTx due to overlapping signs and symptoms. Methods. We analyzed infectious complications and predictors of infection during the early and late periods after CARTx in 213 adult patients with malignancies over a 5-year period. Results. Overall, 75 patients (35%) had at least one infection, mostly within the first 30 days after CARTx. Infections occurring early after CARTx were mostly bacterial, whereas viral infections predominated after Day 30. In multivariate analyses, a high baseline CRP (≥5 mg/dL) was significantly associated with increased risk of infection in the early period after CARTx, and a Day-5-to-baseline-CRP ratio of <1.5 was significantly associated with a higher risk of infection through all 180 days after CARTx and during the late period after CARTx. Conclusions. Infections in adult patients are common up to 180 days after receiving CARTx. High baseline CRP without significant temporal changes may predict risk of infection. Tracking CRP trends may be a useful clinical tool for discerning causes of fever and guiding antibiotic stewardship in this population at high risk for infections.

## Simple Summary

CAR-T cell therapy (CARTx) effectively treats cancer but weakens the immune system, raising infection risk. A key clinical challenge after CARTx is distinguishing infection from cytokine release syndrome (CRS), a common side effect with overlapping symptoms, since both cause fever. This study examined data from 213 adults treated with CARTx to characterize infection patterns and assess whether C-reactive protein (CRP), a simple blood marker, could help predict infection risk. Results showed that 35% of patients developed at least one infection, most occurring within the first 30 days after treatment, with bacterial infections predominating early on. Patients with elevated CRP levels before treatment, or whose CRP failed to rise substantially by day five post-treatment, had a higher risk of infection. These findings suggest CRP tracking could help clinicians determine when antibiotics are genuinely warranted for post-CARTx fevers, potentially improving treatment decisions and reducing unnecessary antibiotic use.

## 1. Introduction

The advent of chimeric antigen receptor-modified T cell immunotherapy (CARTx) for refractory/relapsed hematologic malignancies has produced effective and durable clinical responses [1]. CARTx cell therapies carry the risk of significant toxicities, including cytokine release syndrome (CRS) and immune effector cell-associated neurotoxicity syndrome (ICANS). However, with increased survival rates due to CARTx, non-relapsed mortality (NRM) has emerged as a critical outcome [2]. Long-term follow-up of patients who receive CARTx indicate that infection is a major cause of NRM [2]. This is due, in part, to the lymphodepletive chemotherapy given in preparation for CARTx, which increases the risk for infection [3]. Patients receiving CARTx are also at risk for infection due to (1) the cumulative immunosuppression associated with the underlying malignancy and its prior treatment; (2) the protracted hypogammaglobulinemia resulting from CARTx infusion depleting normal CD19+ B cells and/or long-lived BCMA+ plasma cells; (3) the competitive outgrowth of normal T cells by CARTx, presenting with a leukemia-like picture; and (4) protracted cytopenias [4,5]. The risk of infection ranges anywhere from 30 to 70% after CARTx, and neutropenia can increase the hazard for infections six-fold [6,7]. Moreover, a recent meta-analysis reported that about half of deaths after CARTx are due to infection, exceeding mortality attributable to CRS and ICANS [8]. Therefore, limiting the burden of infection after CARTx is crucial to improving survival among cancer patients [9].

Determining which patients are at risk for infection can be difficult since infection rates in real-world cohorts vary depending on patient characteristics, CARTx cell constructs, follow-up duration, and supportive care practices [2]. While infection risk is affected by both patient- and disease-related factors, it is widely accepted that infection risk is time-dependent and the severity is linked to the evolving immune dysfunction that follows CARTx [2,9,10]. Studies suggest that most infections occur within 30 days after CARTx due to the hematologic nadir following CARTx and lymphodepletion [2,11,12,13,14,15,16,17,18]. Since neutrophil recovery typically begins 2–4 week after CARTx, infections after 30 days and 90 days are fewer and rare, respectively [2,11,12,13,14,15,16,17,18]. Studies also highlight that bacterial infections predominate for the first 30 days after CARTx, whereas viral infections are more frequently seen after 30 days [12,14,15,16,17,19].

In the early period (prior to Day 30) post-CARTx, febrile neutropenia is a common clinical syndrome. This early period is characterized not only by the neutrophil nadir that increases the risk of infection, but also by CRS and ICANS [2]. Therefore, one of the primary challenges for clinicians caring for CARTx patients is differentiating whether febrile neutropenia is caused by infection or a CARTx-related adverse effect, such as CRS. It is crucial to make this distinction promptly, as the treatment for each is significantly different and requires decision-making in a short window to reduce morbidity and mortality. Since there is overlap in the clinical presentation of infection and CARTx-related adverse effects, the focus has recently turned to investigating whether laboratory studies can help discriminate between infectious and non-infectious syndromes. Several studies have examined whether inflammatory biomarkers used commonly in clinical practice, such as C-reactive protein (CRP), can guide distinguishing infection from CRS after CARTx. These studies have implicated that CRP is associated with infection risk in patients receiving CARTx; however, these studies followed patients for only 30–90 days post-CARTx cell infusion, which overlooks the risk of infection caused by prolonged immunosuppression that can afflict CARTx patients [17,20,21,22]. Therefore, we sought to determine the epidemiology of infections and practicality of using CRP to predict infection risk up of 180 days after CARTx, given that the utility of CRP for predicting long-term infection risk beyond day 90 is unknown.

## 2. Materials and Methods

Patients. We performed a single-center retrospective cohort study of adults (age ≥ 18 years) who received CARTx at our institution between March 2018 and March 2023. The study was approved by the University of Maryland Institutional Review Board. Patients were identified through the University of Maryland Immune Effector Cells Database. We included patients who received CD19- (axicabtagene ciloleucel, tisagenlecleucel, brexucabtegene autoleucel, lisocabtagene maraleucel) and BCMA- (idecabtagene vicleucel and ciltacabtagene autoleucel) targeted CARTx.

Cellular therapy clinical protocols. CARTx was administered at the US Food and Drug Administration-approved dose, per institutional protocols. CRS and ICANS were treated according to the American Society of Clinical Oncology guidelines for immune-related adverse events after CARTx [23]. Patients received herpes virus prophylaxis starting the day of CARTx and *Pneumocystis jirovecii* pneumonia (PJP) prophylaxis through Day 180. Patients also received mold prophylaxis based on expert opinion [24]. Patients with febrile neutropenia were treated for infection pre-emptively with anti-Pseudomonal antibiotics.

Definitions and outcomes. The primary outcome was the number of days post-CARTx cell infusion to the first infection. Risk factors for infections and all-cause mortality at Day 180 were secondary outcomes. We defined “Day 0” as the day of CARTx infusion. CRS and ICANS were defined and graded according to each product manufacturer’s protocol.

Infections that started on or prior to Day 0 were not included in the analysis. Infections were defined as bacteremia with a clinically relevant organism (i.e., blood cultures with Coagulase-negative *Staphylococcus* or *Bacillus* spp. were not included), other bacterial infections including pneumonia or UTIs, viral infection, fungal infection (including presumed fungal pneumonia), or *Clostridioides difficile* infection. Included infections were diagnosed by culture isolation from a sterile site (bacteremia or fungemia) or positive PCR. Otherwise, infections were abstracted from clinical documentation by an Infectious Diseases specialist in scenarios where lab results alone were unable to differentiate between true infection and colonization (i.e., UTI) or where the diagnosis of infection is typically based on clinical judgment (i.e., pneumonia, cellulitis) and without culture confirmation.

The time to first infection was defined as the number of days from Day 0 to the diagnosis of infection. If a patient had more than one infection during the follow-up period, only the first infection was included in the analysis. Febrile neutropenia was defined as having a sustained temperature of 38 °C over 1 h or a one-time temperature of 38.3 °C in a patient with an ANC ≤ 500 cells/mm^3^. Early infections were defined as occurring within 1–30 days and late infections were defined as occurring between 31 and 180 days after CARTx cell infusion.

Data collection and statistical analysis. We collected data from Day 0 to Day 180 after CARTx cell infusion. Data collected from the electronic medical record included demographics, malignancy type, history of stem cell transplant and antimicrobial prophylaxis, duration of neutropenia, time to onset of fever after CARTx, type and timing of infections, timing and treatment of CRS or ICANS, CRP levels at Day 0 (baseline CRP) and up to 7 days after CARTx, and mortality up to 180 days after CARTx infusion. Extracted data was collected into an encrypted REDCap database.

Baseline characteristics and outcomes after CARTx cell therapy were reported using summary statistics, such as counts and percentages for categorical variables and medians and interquartile ranges (IQRs) for continuous variables. We assessed whether each categorical variable was associated with infections using Fisher’s exact test. Continuous variables were analyzed using the Wilcoxon test, and time to death was evaluated using the log-rank test. For risk factors for infection, univariate and multivariate logistic regressions were performed. To identify risk factors for infection, univariate logistic regression analyses were initially conducted. Demographic and baseline variables to enter the multivariable logistic regression models were selected based on clinical relevance and whether the risk factors showed a *p* value of <0.05 in the univariate analyses. The Mann–Whitney test was used to assess for significant differences in median CRP values between Day 0 and Day 7 between the groups with and without infection. Fischer’s exact test was used to determine if any associations existed between type of infection (bacteremia, other bacterial infections, *C. difficile*, viral infections, fungal infections) and high baseline CRP (≥5 mg/dL). We used a cutoff of <1.5 for the Day5:baseline CRP ratio based on a prior study; ours was a lower cutoff since the prior study included other markers of inflammation in the score [22]. The log rank test was used for survival curves of time to death. Time to death was defined with surviving patients censored at 180 days. Analyses were performed with SAS Software, Version 9.4. Graph generation was performed in Prism 10, Version 10.2.3.

## 3. Results

### 3.1. Patient Characteristics and Antimicrobial Prophylaxis

Two-hundred and thirteen patients with malignancies were included in the analysis. There were 75 patients (35%) who had at least one infection during the study period. A single patient may have had 1–3 infectious episodes during follow-up. The median number of days to first infection was 16.5 (IQR 4,57). In the cohort, 173 (81%) received CD19- and 40 (19%) received BCMA-CARTx. Detailed patient characteristics are shown in Table 1. The median age was 63 years (IQR 54,70), and most patients were over age 60 (61.5%), male (64%) and White (69%). Forty-seven patients (22%) were African American. The most common indication for CARTx was Diffuse Large B-cell Lymphoma (60%), and most patients had not received a prior stem cell transplant (71%). Colonization with *C. difficile*, MRSA, VRE, or an ESBL organism prior to CARTx was uncommon. There were significantly more patients who were CMV+ who also had infection after CARTx compared to those who were CMV negative.

Details of antimicrobial prophylaxis are listed in Table 1. Most patients were on fluconazole antifungal prophylaxis (53%), while 2.4% and 20% were on voriconazole and posaconazole prophylaxis, respectively. Use of antifungal prophylaxis with micafungin, voriconazole or posaconazole antifungal prophylaxis significantly differed between the groups with and without infection. Most patients were not on CMV prophylaxis (95%), which differed significantly between the groups with and without infection after CARTx.

### 3.2. Infections After CARTx

**Figure 1:**
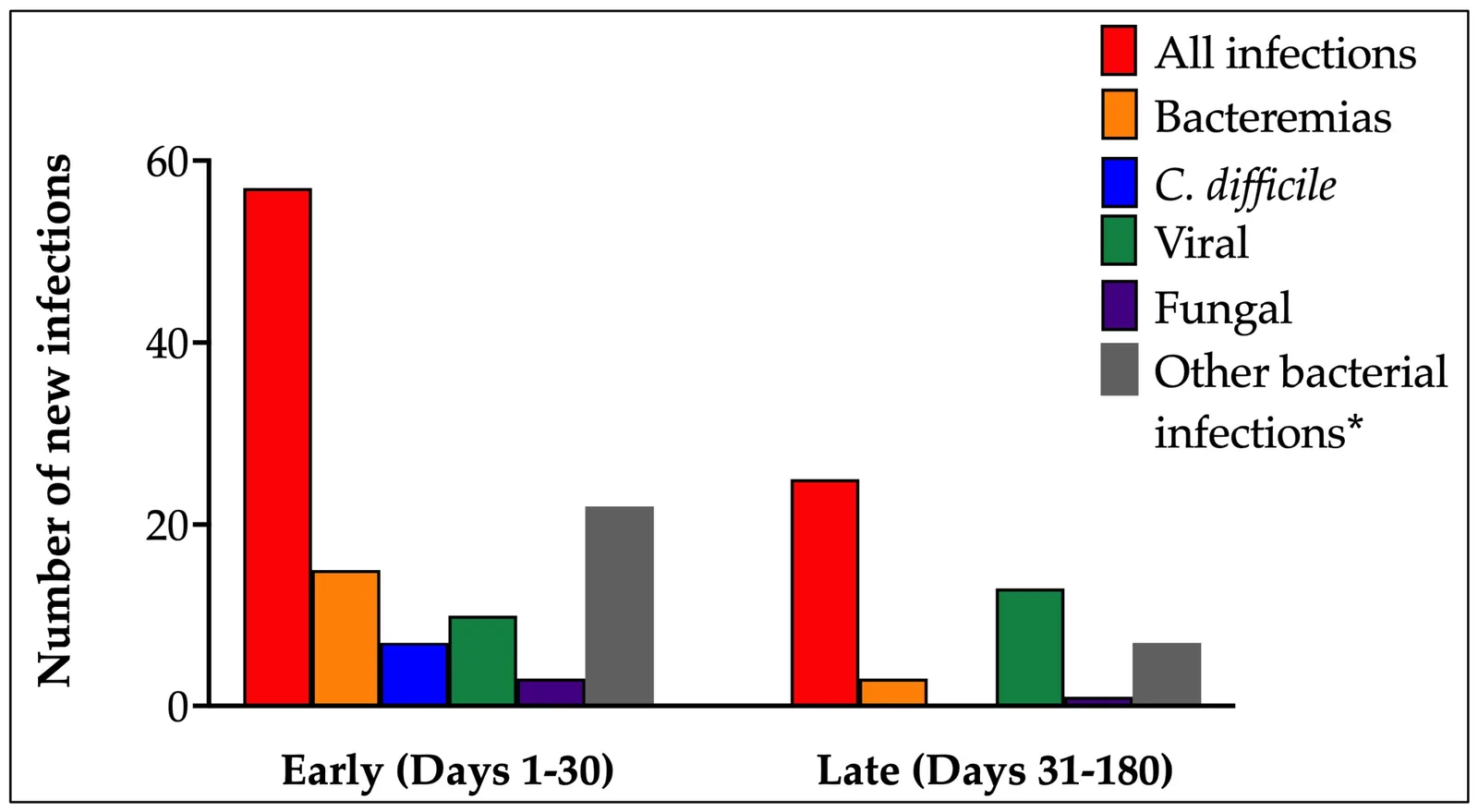
Infections up to 180 days after CARTx. * pneumonia and skin and soft tissue infections. Data is presented as number of episodes of each type of infection and not per patient.

Most infections occurred within 30 days after CARTx (Table 2 and Figure 1). Infections occurring early after CARTx (between Days 1 and 30) were mostly bacterial, whereas viral infections, mainly due to respiratory viruses, predominated after Day 30. There were no significant associations between high baseline CRP (≥5 mg/dL) and type of infection.

### 3.3. Complications of CARTx and Risk Factors Associated with Infections

**Figure 2:**
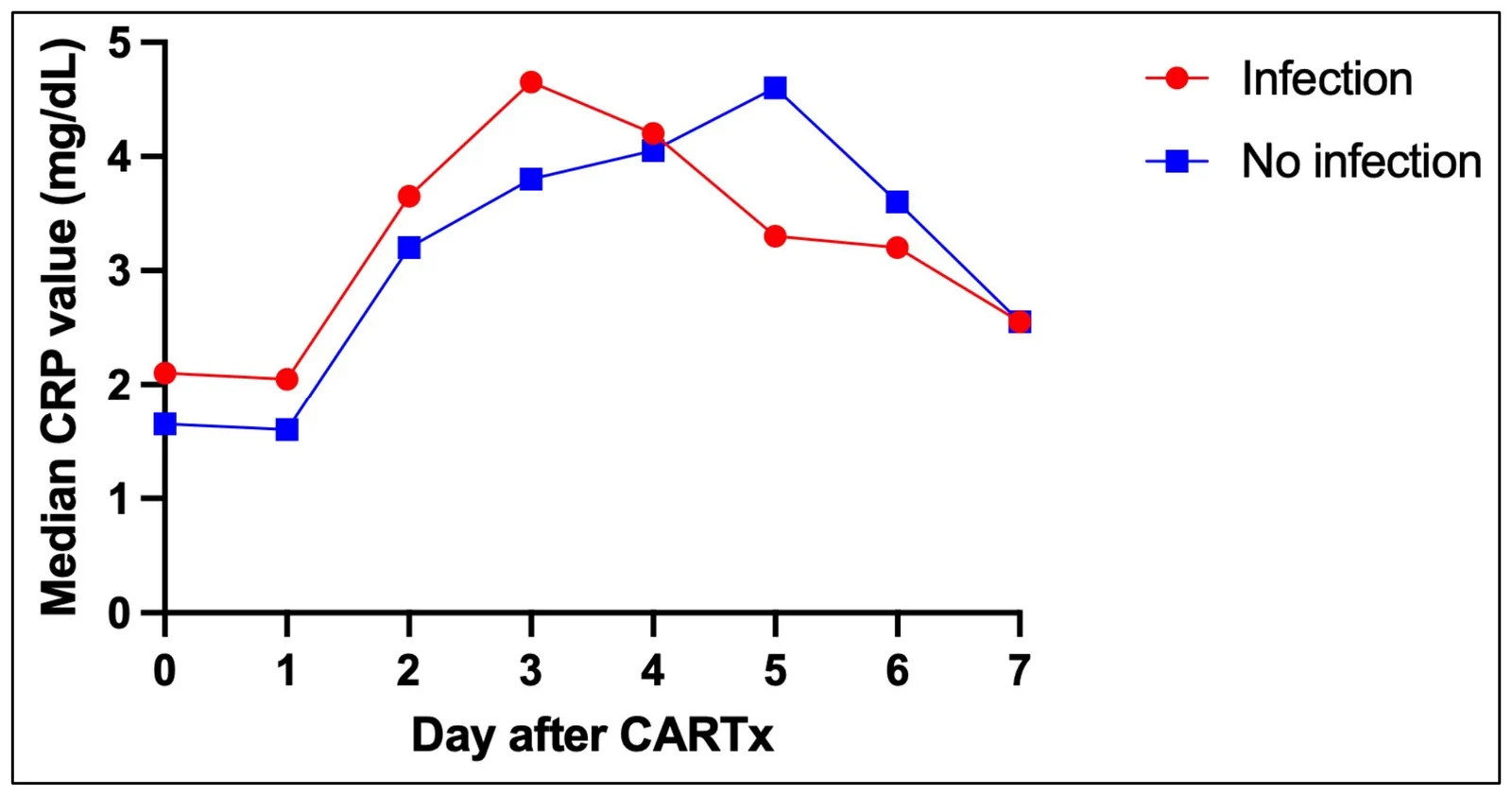
Median CRP (mg/dL) from baseline (Day 0) of CARTx through Day 7 after CARTx in patients with infection (*n* = 75) and without infection (*n* = 138). *p*-value = 0.86 using Mann–Whitney test. CARTx = chimeric antigen receptor-modified T cell immunotherapy. CRP = C-reactive protein.

Details of CARTx complications are shown in Table 1. The median duration of neutropenia was 7 days (IQR 4–10 days) for all patients, and 71% of patients had febrile neutropenia. All 150 patients with febrile neutropenia received anti-pseudomonal antibiotics for treatment. There were 92 patients that had CRS (those with febrile neutropenia but without infection were treated as having CRS). Mortality was 23% at 180 days with a median time to death of 68 days (IQR 45–100 days). The incidence of febrile neutropenia did not differ significantly between patients with and without infection. Most patients had CRS (159, 75%); CRS ≥ grade 2 occurred in 77 (36%) of patients and was significantly associated with infection, but CRS treatment was not. ICANS occurred in 78 (37%) patients, with a grade ≥ 3 significantly associated with infection. Median CRP values from Day 0 to Day 7 after CARTx were similar in the groups with and without infection (Figure 2). Baseline CRP and high baseline CRP did not differ between the groups with and without infection; however, the median ratio of Day 5 to baseline CRP (hereafter referred to as Day 5:baseline) did significantly differ between the groups.

Risk factors for infections were assessed for the entire study period (Table 3) and then assessed for the early and late periods (Table 4). In the multivariate analyses, moderate or severe CRS (grade ≥ 2), CMV+ serostatus, and Day 5: baseline CRP < 1.5 were significantly associated with higher risk of infection. High baseline CRP was significantly associated with infections in the early period, as was steroid plus tocilizumab treatment for CRS in adjusted analyses. For late infections, in adjusted analyses, prior SCT, CMV+ serostatus, and the Day 5:baseline CRP < 1.5 were significant risk factors.

## 4. Discussion

Here, we present data on infectious complications and risk factors for infections occurring within 180 days after CARTx infusion in a large, single-center cohort. The main finding of our study, which adds to prior literature that CRP can discern infection from CRS, was that high baseline CRP and Day 5:baseline CRP < 1.5 are risk factors for infections after CARTx. Our findings imply that a higher baseline CRP, with relatively little increase post-CARTx, may be a useful clinical tool for discerning if fevers are related to infection or CRS. To our knowledge, our real-world cohort includes the highest proportion of African American patients compared to other studies on infection risk after CARTx and includes patients who received both CD19- and BCMA- CARTx.

Patients in our cohort had similar infections post-CARTx compared to prior reports, with most infections occurring in the early phase after CARTx and of bacterial etiology [24,25,26,27]. Concomitantly, mortality was significantly higher in patients with infection. Our findings support several other studies demonstrating that bacterial infections predominate in the early period after CARTx. This period coincides with neutropenia and when immune and host-barrier deficiencies are at their nadir due to conditioning regimens and CRS/ICANS [10,14,15,17,28,29]. However, we did not find that duration of neutropenia increased the risk of infection.

Ours and other reports show that despite the long-term toxicities of cytopenias and hypogammaglobulinemia after CARTx, the incidence of infection after 30 days is lower compared to the early period after CARTx infusion [3]. Our finding of viral infections predominating after Day 30 reflects what is known about immune reconstitution kinetics and is similar to other reports of patients who received CD19- and BCMA-CARTx [13,24,25,28,30,31]. In our cohort, CMV+ serostatus was significantly associated with risk of infections in the late period after CARTx; however, all four CMV infections occurred early after CARTx. Interestingly, all these patients had negative CMV IgG results prior to CARTx, which may have been false negative results secondary to immune dysfunction from prior therapies. In adjusted analyses, we also found that prior SCT and CMV+ serostatus were associated with infection risk in the late period after CARTx. This may have been secondary to the delayed T cell reconstitution and prolonged B cell aplasia that occur as a consequence of cumulative immunosuppression [27,32].

There were very few fungal infections, including invasive mold infections, in our cohort. This is consistent with previous reports in the literature, where invasive fungal disease after CARTx ranged from 0 to 10% [25,28,33]. The low incidence of fungal infections in our cohort may have been related to our prophylaxis protocol. However, in the study by Little et al. of 280 CARTx patients with non-Hodgkin lymphoma not on antifungal prophylaxis, there were also few fungal infections. Our and Little et al.’s findings suggest that fungal infection risk is inherently low in the CARTx population and not affected by use of antifungal prophylaxis [34]. Surprisingly, in our cohort, use of mold prophylaxis significantly differed among patients with and without infection. At our institution, patients are started on mold prophylaxis if they receive high-dose steroids for at least 3 days or a second-line interleukin inhibitor for CRS treatment. Therefore, it is possible that antifungal prophylaxis is serving as a surrogate for immunosuppression caused by CARTx and CRS or ICANS management.

CRS after CARTx is a known risk factor for infections. Severe CRS is associated with delayed neutrophil engraftment, which predisposes to infections; in our cohort, CRS grade ≥2 incidence differed between those with and without infection and increased the risk of infection [10]. Our finding of CRS severity increasing the risk of infection agrees with findings of other studies and multivariate analyses [10,14,15,16,17,24,28]. The mechanism underlying increased infection risk in CRS remains unclear, but it is thought that cytokine storm leads to immune paralysis and compromised mucosal barriers [10]. In addition, the treatment of CRS requires additional immune suppression, further paralyzing the immune system [35]. In the early phase after CARTx, severe infections tend to occur in patients with CRS or ICANS who received immunosuppressing regimens [13,36]. In other studies, ICANS and use of steroids and tocilizumab for CRS treatment were strong predictors of infection after CARTx [28]. In our cohort, the latter was a risk factor for infections in the early period after CARTx.

Since fever is the main sign of and occurs in up to 92% of patients with CRS, it is difficult to discern if fever/febrile neutropenia is from CRS or infection [29]. The majority of our cohort (150, 71%) had febrile neutropenia. All of these patients received empiric anti-pseudomonal antibiotics with their first fever; however, only 58 (39%) had true infection. The high rate of empiric antibiotic use in our study is similar to other reports. In one study, empiric antibiotic was as high as 85.7%, despite a lack of documented bacterial infection [30]. Emerging evidence points to how prudent use of broad-spectrum antibiotics in the peri-CARTx period is crucial, since disruptions in the gut microbiome secondary to antibiotic use affect clinical responses to CARTx [37,38,39,40]. These findings further highlight the need to discern whether fever after CARTx is related to infection or CRS.

There have been a few studies investigating clinical tools to discern the cause of long-term CARTx toxicities. The Endothelial Activation and Stress Index (EASIX) uses creatinine, lactate dehydrogenase, and platelets to identify patients that are at risk for significant toxicities after CARTx but fails to discern if fevers are related to infection or CRS. However, when combined with frailty/performance status, the EASIX has a high NPV for ruling out the risk of early and severe complications [6,41]. In another study of 19 patients, Luo et al. found that in the first 30 days after CARTx, patients with severe CRS (grade ≥ 3) and severe infection (life-threatening or causing death) had significantly higher levels of IL-6 [42]. They mapped the inflammatory signatures of IL-6, IL-8, IL-1Β, and IFN-γ over the first 30 days after CARTx and found that double-peaks of IL-6 were a specific sign for severe infection. They also developed a prediction model using IL-8, IL-1Β, and IFN-γ for diagnosing severe infection for the first 30 days after CARTx.

Though astute, the model is clinically cumbersome since the cytokines in the proposed model are not routinely measured through clinical laboratories and take several days to result. Moreover, the model requires longitudinal data collection, precluding prompt assessment to differentiate CRS from infection.

Another study by Rejeski et al. suggested that CRP can help predict which patients will experience post-CARTx complications [22]. The investigators developed the CAR-HEMATOTOX score, which includes CRP, after associating blood biomarkers with pancytopenia. Calculating the score is feasible since it uses biomarkers routinely included in clinical workup: platelet and absolute neutrophil count, hemoglobin, CRP, and ferritin. The CAR-HEMATOTOX score, like the EASIX, has a high NPV for ruling out the risk of early and severe complications after CARTx, but an added advantage is that the score can risk-stratify patients for severe bacterial infections [6,22,41]. The score includes baseline (measured at Day 5) CRP of ≥3 mg/dL as one of five criteria for patients who are identified as high-risk for bacterial infection. Therefore, the composite score highlights the importance of baseline CRP in predicting the risk of toxicity in CARTx patients, but was not designed to predict the risk of infection alone after CARTx. Our study extends on the CAR-HEMATOTOX score by using CRP but differs in that we assess how one clinically relevant biomarker—CRP—is specifically associated with the risk of infections after CARTx. Our findings also suggest a distinct numeric cutoff for the CRP value, allowing for prompt assessment of infection vs. CRS, which is prudent for the short therapeutic window needed to treat sepsis and prevent septic shock.

Our analysis used a higher baseline CRP than what is in the CAR-HEMATOTOX score; however, the latter includes other markers of inflammation that are accounted for in our study with a higher baseline CRP criterion. Our findings also suggest that Day 5:baseline CRP can be used to stratify those that are at risk for infection overall and in the late period after CARTx, since median Day 5:baseline CRP significantly differed between those with and without infection. Therefore, high baseline CRP and a <1.5-fold increase in the Day 5:baseline CRP are potential clinical tools to discern whether fevers are related to infection in both the early and late periods after CARTx and guide antibiotic de-escalation through all 180 days after CARTx. Use of CRP would be more pragmatic than cytokine-based or composite scores as reported in prior studies, since CRP is a simple clinical marker that is routinely available and interpretable at the bedside.

It is unclear why high baseline CRP with minimal rise predicts risk of infection, but the mechanism may be related to how CRP itself is produced. IL-6 is crucial for CRP production, and since CRS is characterized by the release of a large amount of IL-6, significant increases in CRP have been considered to indicate CRS rather than infection [42,43,44,45]. Another plausible explanation may be that prior to CARTx, there is a smoldering, subacute infection that develops into fulminant infection after CARTx and treatment with immunosuppressants. Last, it is possible that patients with higher infection risk due to immunosuppression may have blunted inflammatory kinetics that do not allow for steep rises in CRP with the advent of infection, explaining why infection is associated with lower rises in CRP.

Our study has a few limitations. First, the retrospective nature with the incorporation of data across years where antimicrobial prophylaxis practices evolved limits interpretation of how prophylaxis impacted infection risk. In addition, we did not determine whether inherent properties of CARTx, such as timing of CRS and CARTx cell expansion, were associated with infection risk. To this end, the relatively small number of late infections may limit the statistical power of the late-period multivariate analysis. The cutoff of the baseline:Day 5 CRP ≤ 1.5 was selected arbitrarily, which makes the results exploratory and requires external validation. Also, our findings may be limited by confounding bias, since patients that had infection were also treated for CRS. Last, although our cohort included patients receiving BCMA-targeting CARTx, where there is a potential for selection bias, we did not include enough patients for a sub-group analysis on how infection risk differs or if there was a difference in CRP trend between these CARTx modalities. For example, among patients receiving CD19- or CD20-CARTx and BCMA-targeting CARTx, there may be differences in CMV reactivation. A few studies that have done stratified analyses on patients receiving BCMA CARTx found higher rates of CMV reactivation in BMCA-CARTx, ranging from 8 to 52.2%, and twice as high as those receiving CD19- and CD20-targeting therapy [46,47,48,49,50,51]. Future studies should include enough patients receiving BCMA-targeting CARTx to evaluate for these differences [46,52,53].

## 5. Conclusions

We found that high baseline CRP and a fold change in CRP of <1.5 are associated with the risk of infection in the early and late periods after CARTx. If our findings are externally validated, then trending CRP could be a pragmatic clinical tool that is easily translatable to clinical practice to assess the need for antimicrobial therapy in patients with fever after CARTx. As more data emerges regarding the relationship between CRP and infection in CARTx patients, further studies should be conducted to understand how fold changes in CRP can be used to develop management strategies for febrile neutropenia and CRS in CARTx patients, providing an excellent opportunity to guide early antibiotic de-escalation and stewardship intervention.

## Figures and Tables

**Table 1:** Patient characteristics and complications of CARTx. ATG = Anti-thymocyte globulin.

	Total N = 213 Patients (%)	Infection N = 75 (%)	No Infection N = 138 (%)	*p*
**Patient Demographics**				
Age at CARTx, median (IQR)	63 (54,70)	61 (53,70)	64 (54,70)	0.31
Age > 60	131 (62)	42 (56)	89 (65)	0.24
Sex				0.55
Male	135 (64)	50 (67)	85 (62)	
Female	78 (37)	25 (33)	53 (38)	
Race				0.92
White	146 (69)	50 (67)	96 (70)	
African American	47 (22)	17 (23)	30 (22)	
Asian/PI	6 (2.8)	2 (2.7)	4 (2.9)	
Other	14 (6.6)	6 (8.0)	8 (5.8)	
Ethnicity				0.58
Hispanic	11 (5.2)	5 (6.7)	6 (4.4)	
Non-Hispanic	200 (94)	69 (92)	131 (95)	
Unknown	2 (0.9)	1 (1.3)	1 (0.72)	
Reason for CARTx				
DLBCL	127 (60)	43 (57)	84 (61)	0.66
Follicular lymphoma	13 (6.1)	6 (8.0)	7 (5.1)	0.39
Mantle cell lymphoma	12 (5.6)	3 (4.0)	9 (6.5)	0.55
Multiple myeloma	40 (19)	16 (21)	24 (17)	0.58
B-cell ALL	11 (5.2)	6 (8.0)	5 (3.6)	0.20
High-grade B-cell lymphoma	4 (1.9)	1 (1.3)	3 (2.2)	1.0
Mediastinal large B-cell lymphoma	4 (1.9)	1 (1.3)	3 (2.2)	1.0
Other	5 (2.3)	2 (2.6)	3 (2.2)	1.0
Prior SCT				0.14
No	151 (71)	47 (63)	104 (75)	
Allogeneic	9 (4.2)	4 (5.3)	5 (3.6)	
Autologous	53 (25)	24 (32)	29 (21)	
Any SCT	62 (29)	28 (37)	34 (25)	0.06
Colonization (within 6 months prior to CARTx)				
*C. difficile*	10 (4.7)	4 (5.3)	6 (4.4)	0.74
MRSA	4 (1.9)	1 (1.3)	3 (2.2)	1.0
VRE	2 (0.9)	2 (2.7)	0	0.13
ESBL	4 (1.9)	2 (2.7)	2 (1.5)	0.61
CMV serostatus				
(R+)	46 (22)	24 (32)	22 (16)	**0.01**
(R−)	31 (15)	10 (13)	21 (15)	0.84
**Prophylaxis**				
Fungal	127 (60)	36 (48)	91 (66)	**0.01**
None	73 (34)	20 (27)	53 (38)	0.09
Fluconazole	112 (53)	42 (56)	70 (51)	0.48
Voriconazole	5 (2.4)	5 (6.7)	0 (0)	**0.01**
Posaconazole	43 (20)	21 (28)	22 (16)	**0.05**
Isavuconazole	9 (4.2)	3 (4.0)	6 (4.4)	1.0
Micafungin	24 (11)	13 (17)	11 (8.0)	**0.04**
PJP				
None	9 (4.2)	2 (2.7)	7 (5.1)	0.50
Trimethoprim-sulfamethoxazole	40 (19)	12 (16)	28 (20)	0.47
Dapsone	9 (4.2)	1 (1.3)	8 (5.8)	0.16
Atovaquone	153 (72)	55 (73)	98 (71)	0.75
Pentamidine	29 (14)	10 (13)	19 (14)	1.0
Hepatitis B	9 (4.2)	4 (5.3)	5 (3.6)	0.72
CMV				
None	202 (95)	67 (89)	135 (98)	**0.02**
Letermovir	6 (2.8)	4 (1.9)	2 (1.5)	0.19
(Val)ganciclovir	2 (0.94)	2 (2.7)	0	0.12
**Complications of CARTx**				
Duration of neutropenia, median days (IQR)	7 (4, 10)	7 (3, 9)	7 (5, 10)	0.94
Febrile neutropenia	150 (71)	58 (78)	92 (68)	0.11
Follow-up time from CARTx median days (IQR)	177 (117,182)	168 (86,180)	180 (139, 183)	**0.01**
Mortality at 180 days	49 (23)	24 (32)	25 (18)	**0.03**
Time to death, median days (IQR)	68 (45,100)	180 (94,180)	180 (180, 180)	**0.02**
CRS				0.12
CRS ≥ grade 2	77 (36)	35 (47)	42 (30)	**0.02**
CRS ≥ grade 3	8 (3.8)	4 (5.3)	4 (2.9)	0.45
ICANS				0.09
ICANS ≥ 2	48 (23)	21 (28)	27 (20)	0.17
ICANS ≥ 3	28 (13)	15 (20)	13 (9.4)	**0.03**
CRP				
Baseline CRP, median (IQR)	1.9 (0.8, 4.2)	2 (1, 5.7)	1.7 (0.7,3.7)	0.22
High baseline CRP (≥5 mg/dL)	72 (34)	28 (37)	44 (32)	0.45
Day 5 CRP: baseline CRP, median (IQR)	1.6 (0.7, 4.4)	1.0 (0.5, 4.0)	1.9 (1.0, 5.3)	**0.01**
Day 5 CRP:baseline <1.5	111 (52)	46 (61)	66 (47)	0.06

Bolded *p*-values indicate statistical significance.

**Table 2:** Infections occurring early and late after CARTx.

	Early Infections, No. (Days 0–30)	Late Infections, No. (Days 31–180)
All infections	57	24
Bacteremia	15	3
Gram-negative	7	2
Gram-positive	8	1
*C. difficile* infection	7	0
Viral infection	10	14
Adenovirus	1	1
BK virus	-	1
CMV	4	-
HHV-6	-	1
Parainfluenza	1	2
Parvovirus B19	-	1
Rhinovirus/enterovirus	3	2
RSV	-	1
SARS-CoV-2	1	4
Fungal infection	3	1
Other bacterial infections	22	7

**Table 3:** Risk factors for infections. SCT = Stem cell transplant. CRS = Cytokine release syndrome. ICANS = Immune-effector cell-associated neurotoxicity syndrome. CMV = Cytomegalovirus. CRP = C-reactive protein.

	Univariate		Multivariate	
	Odds Ratio (95% CI)	*p*	Odds Ratio (95% CI)	*p*
Age > 60	0.70 (0.4, 1.2)	0.22	0.86 (0.45, 1.6)	0.65
Prior SCT	1.8 (1.0, 3.34)	**0.05**	1.8 (0.91, 3.5)	0.09
CRS ≥ grade 2	2.0 (1.1, 3.6)	**0.02**	2.3 (1.2, 4.5)	**0.01**
ICANS ≥ 3	2.4 (1.1, 5.4)	**0.03**	2.0 (0.77, 5.0)	0.16
No CMV prophylaxis	0.19 (0.05, 0.72)	**0.02**	0.25 (0.06, 1.06)	0.06
CMV serostatus (R+)	2.48 (1.3, 4.8)	**0.01**	2.4 (1.1, 5.0)	**0.02**
High baseline CRP (≥5 mg/dL)	1.28 (0.71, 2.3)	0.42	1.01 (0.46, 2.2)	0.99
Day 5 CRP:baseline CRP < 1.5	1.78 (1.0, 3.2)	**0.05**	2.04 (1.05, 3.96)	**0.04**

Bolded *p* values indicate statistical significance.

**Table 4:** Risk factors for early or late infection among patients undergoing CARTx. SCT = Stem cell transplant. CMV = Cytomegalovirus. CRS = Cytokine release syndrome. ICANS = Immune-effector cell-associated neurotoxicity syndrome.

	Early Infection (N = 52 Patients)				Late Infection (N = 23 Patients)			
	Unadjusted OR (95% CI)	*p*	Adjusted OR (95% CI)	*p*	Unadjusted OR (95% CI)	*p*	Adjusted OR (95% CI)	*p*
Age > 60	1.7 (0.90, 3.2)	0.10	0.57 (0.29, 1.1)	0.10	0.96 (0.38 2.45)	0.94	1.3 (0.45, 3.9)	0.61
Male Sex	1.2 (0.65, 2.3)	0.51	--	--	0.34 (0.11, 1.0)	0.06	0.19 (0.05, 0.71)	**0.01**
Prior SCT	1.3 (0.63, 2.5)	0.51	--	--	2.8 (1.1, 6.9)	**0.03**	3.8 (1.3, 11.1)	**0.01**
CMV serostatus (R+)	1.49 (0.72, 3.06)	0.28	--	--	4.0 (1.6, 10.4)	**0.01**	4.0 (1.3, 11.1)	**0.01**
No CMV prophylaxis	0.36 (0.11, 1.2)	0.10	--	--	0.15 (0.03, 0.79)	**0.03**	0.09 (0.01, 0.69)	**0.02**
Steroid plus tocilizumab for CRS (ref: no treatment)	2.9 (1.4 5.9)	**0.01**	2.5 (1.2, 5.3)	**0.02**	0.51 (0.21, 1.26)	0.14	--	--
CRS ≥ grade 2	2.4 (1.3, 4.5)	**0.01**	--	--	1.2(0.48, 3.1)	0.68	--	--
ICANS ≥ 3	2.7 (1.2, 6.2)	**0.02**	2.0 (0.82, 4.7)	0.13	1.4 (0.38, 5.5)	0.60	--	--
High baseline CRP (≥5 mg/dL)	2.6 (1.3, 5.3)	**0.01**	2.2 (1.0, 4.5)	**0.04**	0.71 (0.2, 2.6)	0.61	0.33 (0.08, 1.4)	0.14
Day 5:baseline CRP <1.5	1.3 (0.72, 2.53)	0.36	--	--	2.6 (1.0, 6.6)	**0.05**	4.4 (1.4, 13.7)	**0.01**

Bolded *p* values indicate statistical significance.

## Data Availability

Data are contained within the article.

## Abbreviations

The following abbreviations are used in this manuscript:

CARTx	Chimeric antigen receptor-modified T cell immunotherapy
CRP	C-reactive protein
CRS	Cytokine release syndrome
ICANS	Immune effector cell-associated neurotoxicity syndrome
IQR	Interquartile range
PJP	*Pneumocystis jirovecii* pneumonia
